# Acute Cardiovascular Effects of Hydrotreated Vegetable Oil Exhaust

**DOI:** 10.3389/fphys.2022.828311

**Published:** 2022-03-08

**Authors:** Youna Marc-Derrien, Louise Gren, Katrin Dierschke, Maria Albin, Anders Gudmundsson, Aneta Wierzbicka, Frida Sandberg

**Affiliations:** ^1^Department of Biomedical Engineering, Lund University, Lund, Sweden; ^2^Ergonomics and Aerosol Technology, Lund University, Lund, Sweden; ^3^Division of Occupational and Environmental Medicine, Lund University, Lund, Sweden; ^4^Unit of Occupational Medicine, Institute of Environmental Medicine, Karolinska Institutet, Stockholm, Sweden

**Keywords:** pulse decomposition analysis, PPG (photoplethysmography), HRV (heart rate variability), pulse transit time (PTT), air pollution, chamber study, hydrotreated vegetable oil (HVO), biomedical signal processing

## Abstract

Ambient air pollution is recognized as a key risk factor for cardiovascular morbidity and mortality contributing to the global disease burden. The use of renewable diesel fuels, such as hydrotreated vegetable oil (HVO), have increased in recent years and its impact on human health are not completely known. The present study investigated changes in cardiovascular tone in response to exposure to diluted HVO exhaust. The study participants, 19 healthy volunteers, were exposed in a chamber on four separate occasions for 3 h and in a randomized order to: (1) HVO exhaust from a wheel loader without exhaust aftertreatment, (2) HVO exhaust from a wheel loader with an aftertreatment system, (3) clean air enriched with dry NaCl salt particles, and (4) clean air. Synchronized electrocardiogram (ECG) and photoplethysmogram (PPG) signals were recorded throughout the exposure sessions. Pulse decomposition analysis (PDA) was applied to characterize PPG pulse morphology, and heart rate variability (HRV) indexes as well as pulse transit time (PTT) indexes were computed. Relative changes of PDA features, HRV features and PTT features at 1, 2, and 3 h after onset of the exposure was obtained for each participant and exposure session. The PDA index A_13_, reflecting vascular compliance, increased significantly in both HVO exposure sessions but not in the clean air or NaCl exposure sessions. However, the individual variation was large and the differences between exposure sessions were not statistically significant.

## 1. Introduction

Ambient air pollution is recognized as a key risk factor influencing the global disease burden (Landrigan et al., 2018). Both long-term and short-term exposure to particulate matter (PM) air pollution has been shown to contribute to cardiovascular morbidity and mortality (Franklin et al., 2015). Exposure to PM air pollution correlates with subclinical pathologies underlying cardiovascular disease, including systemic inflammation and oxidative stress, atherosclerosis, thrombosis, endothelial dysfunction, hypertension, cardiac remodeling, and arrhythmia (Hamanaka and Mutlu, 2018). The biological mechanisms behind PM-induced cardiovascular disease are not completely known. One of the suggested mechanisms is that inhaled PM acts on sensory receptors in the lung, promoting activation of the hypothalamic pituitary adrenal (HPA) axis resulting in increased sympathetic activity in the autonomic nervous system (ANS) (Hamanaka and Mutlu, 2018).

Heart rate variability (HRV) analysis is widely used to characterize the functions of the ANS (Task Force, 1996; Sassi et al., 2015). Previous studies have linked PM_2.5_ exposure to decreased HRV (Pieters et al., 2012), however, results from controlled exposure studies are inconsistent. Hemmingsen et al. (2015) found that HRV significantly decreased during exposure to real-life levels of particles from urban streets in an exposure chamber in a study population of overweight, older adults. In contrast, Mills et al. (2011a) found that brief exposure to diluted diesel exhaust did not alter HRV in healthy volunteers or well-treated patients with stable coronary heart disease.

Previous studies have found an association between air pollution and arterial stiffness (Zanoli et al., 2017). Moreover, results from controlled exposure studies suggest that short-term exposure to air pollution are associated with changes in vascular tone. Lucking et al. (2011) and Mills et al. (2011b) have reported impairment of vasomotor responses to endothelium-dependent and endothelium independent vasodilators after diesel exhaust exposure. Transient increases in arterial stiffness have been reported in response to exposure to diluted diesel exhaust (Lundbäck et al., 2009), and diluted wood smoke (Unosson et al., 2013), respectively. In those studies, arterial stiffness was assessed with applanation tonometry. The pulses at the carotid and femoral arteries were used to estimate carotid-femoral pulse wave velocity (PWV) which is regarded as gold standard to assess arterial stiffness (Laurent et al., 2006). Further, the pulse obtained at the radial artery was used to estimate an aortic pressure pulse from which an augmentation index quantifying the ration of the amplitude of the reflected pressure wave to the pulse pressure peaks was derived.

Photoplethysmography (PPG) is used as inexpensive and operator independent alternative to applanation tonometry (Millasseau et al., 2006; von Wowern et al., 2015). The PPG pulse transit time (PTT), commonly defined as the time interval between the ECG R peak and the corresponding PPG pulse onset, is inversely related to PWV and is used as a marker of arterial compliance. Several different indexes characterizing the PPG finger pulse morphology have been proposed to quantify arterial stiffness and vascular tone (Allen, 2007). Most of these indexes are computed based on peak detection in the PPG signal and its first and second derivative( Elgendi, 2012), which is very sensitive to noise and deviating pulse morphology and to remedy this, more robust techniques based on PPG pulse decomposition have been proposed. One such method is the pulse decomposition analysis (PDA) recently proposed for PPG based assessment of changes in vascular tone in response to mental stress by Kontaxis et al. (2021), which we use in the present study.

Diesel exhaust emission is considered one of the main sources of air pollution in cities. Diesel exhaust is a complex mixture of PM and gases, including elemental carbon (EC), carbon monoxide (CO), carbon dioxide (CO_2_), nitric oxide (NO), nitrogen dioxide (NO_2_), hydrocarbons (HC), and polyaromatic hydrocarbons (PAHs). The use of renewable diesel fuels, such as hydrotreated vegetable oil (HVO), have increased in recent years to reduce CO_2_ emissions from diesel vehicles. Hydrotreated vegetable oil generate exhaust emission similar to diesel exhaust in composition, but can reduce the PM emissions by 20–50% compared to petroleum diesel (Kuronen et al., 2007; Murtonen et al., 2010). In order to reduce the environmental and health hazardous emission from diesel vehicles, emission abatement techniques such aftertreatment systems have been enforced in newer vehicles. Such systems are located in the exhaust pipe and can for example contain a diesel oxidation catalyst for removing CO and organic compounds (Zeraati-Rezaei et al., 2020), and a diesel particulate filter (DPF) which can remove a large fraction of the PM by oxidizing the soot particles (Reşitoĝlu and K Altinişik K, 2015). The impact of such difference in exhaust composition on human health are not completely known (Landwehr et al., 2021).

The purpose of the present study is to investigate changes in HRV and vascular tone in response to diluted HVO exhaust with and without exhaust particles in a controlled exposure chamber study. We analyze PPG pulse morphology, HRV, and PTT in synchronized ECG and PPG signals from 19 healthy volunteers in a randomized double-blinded cross-over study that compare four exposure scenarios; HVO exhaust from a wheel loader without exhaust aftertreatment, HVO exhaust from a wheel loader with an aftertreatment system, clean air, and clean air enriched with dry NaCl salt particles.

## 2. Materials

The exposures were conducted in a 22 m^3^ stainless steel chamber with an air exchange rate of 4 changes per hour at the Aerosol Laboratory, Lund University, Sweden, during fall 2019. The study population consisted of 19 healthy volunteers (10 males, age 20–55 years) which complied with the inclusion criteria: no symptoms or diagnosis of lung disease or asthma; a normal standard ECG reading; no allergy- or cardiovascular medication; non-smokers the last three years. The study participants were instructed to avoid caffeine in the morning and alcohol for 24 h before each exposure session. Further, the participants were told to try to live as normal and similar as possible before each exposure session. Previous studies have investigated pulmonary function and self-rated symptoms (Gren et al., 2022), urinary PAH metabolites and biomarkers (Krais et al., 2021), and genotoxic responses (Scholten et al., 2021), respectively, in response to the exposures.

The participants were exposed on separate occasions for 3 h in a randomized order to the following exposure scenarios: filtered air (FA, PM ≃ 1 μg/m^3^), filtered air with dry salt NaCl particles (NaCl, ≈120μ*gm*^−3^ of PM_2.5_), HVO exhaust from a wheel loader with an aftertreatment system, i.e., emission with nitrogen oxides and low amounts of particulate matter (HVO_NOx_, PM ≃ 1 μg/m^3^, NO = 2.0 ppm, NO_2_ = 0.7 ppm), and HVO exhaust from a wheel loader without aftertreatment, i.e., emission with nitrogen oxides and particulate matter (HVO_NOx+PM_, PM ≃ 90 μg/m^3^, EC = 54 μg/m^3^, NO = 3.4 ppm, NO_2_ = 0.6 ppm). The temperature was kept at 26 ± 1°C and the relative humidity at 33 ± 4%. The duration of the exposures was limited to 3 h for practical reasons to allow for controlled conditions; during this time the participants did not leave the chamber.

The HVO_NOx+PM_ and HVO_NOx_ exhaust was generated with a smaller vehicle (without the aftertreatment system) following the emission standard Euro IIIa for non-road engines, and a larger vehicle (with the aftertreatment system) following the emission standard Euro V, respectively. The vehicles were run on 100% HVO and the exhaust was extracted from the exhaust pipe, transported in heated tubing and diluted in two steps to a total dilution ratio of 1:160. The supply air for the dilution was filtered from particles with a high efficiency particulate absorbing (HEPA) filter and from gases with an active carbon filter. The resulting exposure concentrations complied with current EU occupational exposure limits (OELs) of NO, NO_2_, formaldehyde, PAHs, and future OEL of elemental carbon (2023). The NaCl exposure was generated by nebulizing a salt solution (chemical-grade NaCl dissolved in ultrapure water) with a constant output atomizer model 3076 (TSI Inc. Shoreview, MN, U.S.A.) together with a custom-built nebulizer. The aerosol was dried with a dilution flow of HEPA-filtered air in a 10 L steel chamber, and the relative humidity was kept was kept below 30% in this step (except during one exposure that had <40% RH), to ensure that the salt particles were in dry solid form. The exposure setup and the HVO exhaust generation are described in detail in Gren et al. (2022).

Synchronized PPG and ECG recordings were acquired using a custom-made medical device, CardioHolter v6.2, developed at Kaunas University of Technology, Lithuania. Three-lead ECG was continuously recorded during the experimental protocol at a sampling frequency of 1 kHz. Transmittance PPG at wavelengths of 635 nm (IR) and 960 nm (R) from the right and left hand index fingers, sampled at 250 Hz were recorded during four 15-min sessions when the participants were told to rest: pre-exposure and after 1, 2, and 3 h into the exposure, respectively.

The study was conducted in accordance with the Declaration of Helsinki and approved by the Swedish Ethical Review Authority (registration no. 2019-03320). All participants gave their informed consent before the experiment.

## 3. Methods

### 3.1. PPG Pulse Waveform Analysis

#### 3.1.1. PPG Pre-processing and Pulse Detection

The PPG signals are subjected to high-pass filtering for the purpose of baseline removal using a 2nd order Butterworth filter with cut-off frequency of 0.5 Hz, and interpolated to 1 kHz using cubic splines to match the temporal resolution of corresponding ECG signals. Segments of the PPG signal containing large amplitude artifacts are excluded from further analysis; such intervals are identified using the energy-related approach proposed by Armañac et al. (2019); if the 2-s moving variance of the squared PPG signal *y*^2^(*n*) is more than 4 times larger than the 100-s moving median of *y*^2^(*n*), the sample *y*(*n*) is excluded from further analysis. Finally, the PPG signals are subjected to low-pass filtering at 10 Hz to attenuate high-frequency noise; *x*_PPG_(*n*) denotes the pre-processed PPG signal.

Detection of PPG pulse onset times is carried out using the method proposed by Argüello-Prada (2019), which is based on the first derivative of *x*_PPG_(*n*), denoted $x_{\text{PPG}}^{\prime }\left(n\right)$. The maximum of $x_{\text{PPG}}^{\prime }\left(n\right)$ associated with the *i*^th^ PPG pulse is denoted *n*_F_(*i*). The pulse onset *n*_B_(*i*) is defined as:


(1) $$\begin{array}{r} n_{\text{B}}\left(i\right)=\arg\underset{n\in \Omega }{\min}\left\{\left|x_{\text{PPG}}^{\prime }\left(n\right)-0.05\cdot x_{\text{PPG}}^{\prime }\left(n_{\text{F}}\left(i\right)\right)\right|\right\} \end{array}$$

where Ω = [*n*_F_(*i*)−0.3·F_s_, *n*_F_(*i*)], and *F*_*s*_ = 1000 Hz is the sampling rate of *x*_PPG_(*n*). In other words, *n*_B_(*i*) is the point in an interval 300 ms prior to *n*_F_(*i*) where the slope of *x*_PPG_(*n*) has been reduced by 5%.

Further, linear interpolation of *x*_PPG_(*n*_B_(*i*)) is subtracted from *x*_PPG_(*n*), yielding ${\overset{ˇ}{x}}_{\text{PPG}}\left(n\right)$, so that each pulse begins and ends with zero amplitude. The *i*^th^ PPG pulse is defined as


(2) $$x_{i}(n)={\overset{ˇ}{x}}_{\text{PPG}}(n),\;n\in \left[n_{\text{B}(i)},n_{\text{B}(i+1)}\right]$$

PPG pulses immediately preceding and following each detected artefact segment are excluded from further analysis.

#### 3.1.2. Pulse Decomposition Analysis

A pulse decomposition analysis (PDA) technique adapted from Kontaxis et al. (2021) is applied for PPG pulse waveform modeling. Each pulse *x*_*i*_(*n*) is decomposed into *K* symmetrical waves: *x*_*i*, 1_(*n*), ..., *x*_*i,K*_(*n*) and a residual signal. In this study, *K* is set to 5. The up-slope interval of the running residual ${\tilde{x}}_{i,j}\left(n\right)$ is defined, with its onset denoted as *n*_O_*j*__ and its end as *n*_E_*j*__. The point *n*_O_*j*__ corresponds to of the first non-negative sample and *n*_E_*j*__ to the position of the first relative maximum of ${\tilde{x}}_{i,j}\left(n\right)$. The inner wave is obtained by concatenating the up-slope of the residual with itself horizontally flipped:


(3) $$x_{i,j}(n)=\left\{ \begin{array}{l} {\tilde{x}}_{i,j}(n),\;n\in \left[n_{{\text{O}}_{j}},n_{{\text{E}}_{j}}\right]\;\\ {\tilde{x}}_{i,j}(-n+2\cdot n_{{\text{E}}_{j}}),\;n\in \left[n_{{\text{E}}_{j}},2\cdot n_{{\text{E}}_{j}}-n_{{\text{O}}_{j}}\right]\;\\ 0,\text{ otherwise} \end{array}\right.$$

The *j*th inner wave is then subtracted from the running residual, i.e., ${\tilde{x}}_{i,j+1}\left(n\right)={\tilde{x}}_{i,j}\left(n\right)-x_{i,j}\left(n\right)$ and the following inner waves are computed recursively. A minimum amplitude threshold is set: an inner wave is defined only if ${\tilde{x}}_{i,j}\left(n_{{\text{E}}_{j}}\right)$ exceeds γ = 0.05 ·max{*x*_*i*_(*n*)}. Moreover, either the presence of a relative maximum with amplitude lower than γ, or a slope change which, however, does not appear as a relative maximum in ${\tilde{x}}_{i,j}\left(n\right)$ can lead to erroneous pulse decomposition. The point *n*_O_*j*__ is thus redefined as the next sample after the last negative-amplitude sample of ${\tilde{x}}_{i,j}^{\prime }\left(n\right)$ and *n*_E_*j*__ as the position of the first relative minimum of ${\tilde{x}}_{i,j}^{\prime }\left(n\right)$ for which ${\tilde{x}}_{i,j}\left(n_{{\text{E}}_{j}}\right)\geq \gamma$ is fulfilled.

#### 3.1.3. Pulse Waveform Features

**Figure 1:**
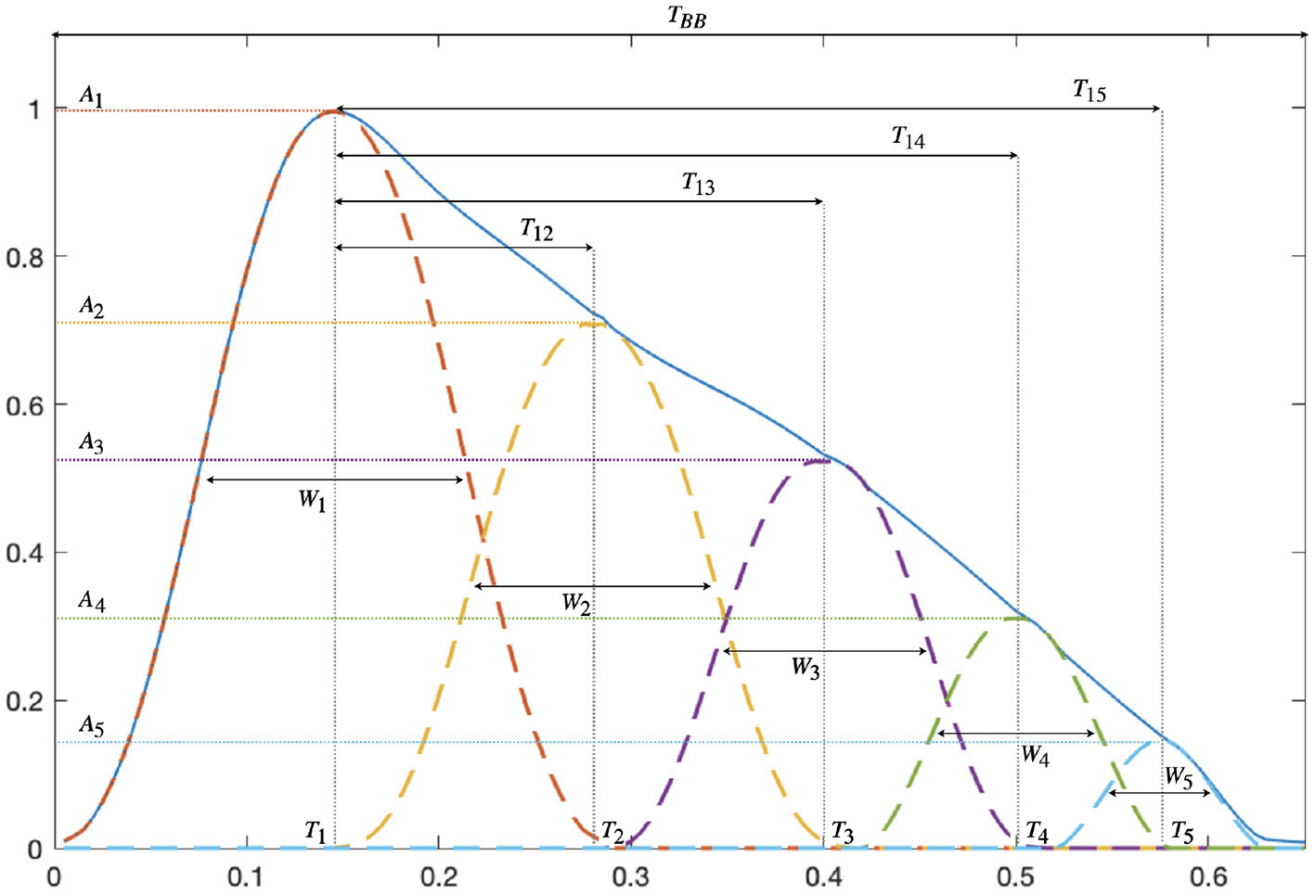
PPG pulse waveform features derived from the amplitude A_*j*_, position T_*j*_, and width W_*j*_ of the *K* = 5 decomposed waves.

Morphological attributes related to vascular compliance properties are extracted from each modeled PPG pulse. For every inner wave, A_*j*_(*i*) and T_*j*_(*i*) are, respectively, defined as the amplitude and position of the absolute maximum of *x*_*i,j*_(*n*). The width of each inner wave, denoted as W_*j*_(*i*) is estimated by the width at half-maximum of *x*_*i,j*_(*n*). Figure 1 shows an example of pulse waveform characteristics.

The time delay T_1*j*_(*i*), as well as the percentage of amplitude loss A_1*j*_(*i*) between the first and the *j*th wave, are calculated:


(4) $$\begin{array}{r} {\text{T}}_{1j}\left(i\right)={\text{T}}_{j}\left(i\right)-{\text{T}}_{1}\left(i\right)\;\text{and }{\text{A}}_{1j}\left(i\right)=\frac{{\text{A}}_{j}\left(i\right)-{\text{A}}_{1}\left(i\right)}{{\text{A}}_{1}\left(i\right)}\times 100,\\ \;j\in \left[2,5\right] \end{array}$$

A pulse is considered distorted if one of the following criteria is fulfilled: (a) the pulse is decomposed into less than five waves, (b) the amplitude of the main wave is not the largest of the five waves, (c) the second wave is located at the end of the pulse interval, i.e., T_2_(*i*)>0.8·T_BB_(*i*), or (d) the fifth wave is located at the beginning of the pulse interval, i.e., T_5_(*i*) <0.4·T_BB_(*i*). All distorted pulses are excluded from further analysis.

PDA-derived features are computed for each pulse in the last 5-min segment of each 15-min PPG recording. For each study participant, the median value and the median absolute deviation (MAD) of each pulse waveform characteristic is calculated, separately for every exposure scenario (FA, NaCl, HVO_NOx_, HVO_NOx+PM_) and time into exposure (pre-exposure and after 1, 2, and 3 h). Complementary outlier rejection is performed; if a feature value deviates more than 5· MAD from the median the it is excluded, and the median is updated.

### 3.2. Heart Rate Variability Analysis

Classical time-domain and frequency-domain HRV features are calculated based on ECG segments from the last 5 min of rest pre-exposure and after 1, 2, and 3 h into the exposure, respectively, i.e., the same 5 min window that are used for PPG waveform analysis.

Following detection of R-peaks in the ECG, beats are clustered and ectopic beats are identified based on heartbeat morphology (Lagerholm et al., 2000). For each ECG recording an RR interval series is constructed


(5) $$\begin{array}{r} d_{\text{RR}}\left(t_{\text{E}}\left(i\right)\right)=t_{\text{E}}\left(i\right)-t_{\text{E}}\left(i-1\right) \end{array}$$

where *t*_E_(*i*) denotes the time of the *i*th R-peak. RR intervals preceding and following ectopic beats, and RR intervals deviating more than 20% from the mean of the 50 preceding RR intervals are excluded from the series to obtain a normal-to-normal interval series *d*_NN_.

The following time-domain HRV features are obtained directly from *d*_NN_(*t*_E_(*j*)): mean normal-to-normal interval (NN), standard deviation of NN intervals (SDNN), root mean square of successive differences of adjacent NN intervals (rMSSD), and standard deviation of adjacent NN interval differences (SDSD).

To calculate the frequency-domain HRV features *d*_NN_ is uniformly re-sampled to 4 Hz using cubic spline interpolation and the fast Fourier transform is applied. The power in the low-frequency band (LF, 0.04–0.15 Hz) and in the high frequency band (HF, 0.15–0.4 Hz) are estimated, as well as the LF to HF ratio (LF/HF) and the relative power in the LF and HF bands to the total power (HF norm and LF norm).

### 3.3. Pulse Transit Time Analysis

Pulse transit time are calculated based on synchronized PPG and ECG segments from the last 5 min of rest pre-exposure and after 1, 2, and 3 h into the exposure, respectively, i.e., the same 5 min window that are used for HRV and PPG waveform analysis.

The pulse transit time interval series is computed as


(6) $$\begin{array}{r} d_{\text{PTT}}\left(i\right)=t_{\text{P}}\left(i\right)-t_{\text{E}}\left(i\right) \end{array}$$

where *t*_P_(*i*) denotes the PPG pulse onset time, given by the *n*_B_ [cf. Equation (1)] that occurs within the interval [*t*_E_(*i*), *t*_E_(*i*+1)].

PTT intervals deviating more than 30% from the mean length of the 50 preceding intervals are considered abnormal and excluded from further analysis. Classical time domain indices are measured: mean PTT interval (PTT), standard deviation of PTT intervals (SDPTT), root mean square of successive differences of adjacent PTT intervals (rMSSD^PTT^), and standard deviation of adjacent PTT interval differences (SDSD^PTT^).

### 3.4. Statistical Analysis

For each participant *i* the percentage change in feature $F_{i}$ a given time point (*t* = 1, 2, and 3 h) after exposure onset, relative to pre-exposure parameter, $F_{i}^{0}$, is computed:


(7) $$\begin{array}{r} \Delta {\left(F_{i}\right)}^{\text{t}}=\frac{F_{i}^{\text{t}}-F_{i}^{\text{0}}}{F_{i}^{\text{0}}}\times 100 \end{array}$$

For each exposure and time into exposure *t*, a Wilcoxon signed rank test is applied to test if the participant median $\Delta {\left(F_{i}\right)}^{\text{t}}$ differs significantly from zero; for the PDA and PTT features, $\Delta {\left(F_{i}\right)}^{\text{t}}$ is averaged over PPG channels prior to this test.

Further, a non-parametric Friedman one-way repeated measure analysis of variance by ranks is used to determine if the exposure type (FA, NaCl, HVO_NOx_, HVO_NOx+PM_) effects $\Delta {\left(F_{i}\right)}^{\text{t}}$. For the PDA and PTT features, $\Delta {\left(F_{i}\right)}^{\text{t}}$ obtained from the different PPG channels is considered as repeated observations. A multi-comparison test (*post-hoc* analysis) with Bonferroni applied to determine if differences between exposure types are significant.

## 4. Results

### 4.1. Data Acquisition and Experimental Protocol

A total of 18 study participants completed the whole experimental protocol; one participant only attended sessions NaCl and HVO_NOx_. PPG recordings were partly missing and/or of insufficient quality for one participant during exposure NaCl and two participants during exposure HVO_NOx+PM_. At 3 h after exposure onset, PDA features could be obtained from 18 (FA), 17 (NaCl), 19 (HVO_NOx_), and 17 (HVO_NOx+PM_), participants, respectively.

ECG recordings were partly missing from three participants during exposure NaCl, two participants during exposure HVO_NOx_ and two participants during exposure HVO_NOx+PM_. At 3 h after exposure onset, HRV features could be obtained from 18 (FA), 16 (NaCl), 19 (HVO_NOx_), 16 (HVO_NOx+PM_) and participants, respectively. Further, at 3 h after exposure onset, PTT features could be obtained from 18 (FA), 16 (NaCl), 16 (HVO_NOx_), and 18 (HVO_NOx+PM_) participants, respectively.

Changes in PDA features at 3 h after exposure for all exposure sessions could be obtained from 15 participants, and changes in HRV and PTT features at 3 h after exposure for all exposure sessions could be obtained from 13 participants; the comparison between exposure sessions is based on this subset of data.

### 4.2. PPG Pulse Characteristics

For each exposure scenario, the percentage of discarded pulses (mean±std) is 4.9 ± 9.3% (FA), 4.4 ± 7.7% (NaCl), 4.8 ± 8.1% (HVO_NOx_), and 4.5 ± 9.1% (HVO_NOx+PM_). These results suggest that few distorted pulses are present in PPG signal recordings in average, but the percentages varies between recordings. The number of analyzed pulses (mean±std) in each 5-min segment is 206 ± 114 (FA), 212 ± 109 (NaCl), 204 ± 105 (HVO_NOx_), and 195 ± 118 (HVO_NOx+PM_), which is considered sufficient for robust estimation of the PDA features (cf. Section 3.1.3).

On average, pre-exposure PPG pulse characteristics did not differ significantly between the exposure sessions (Table 1). A large range of responses was however observed for each participant and feature individually, justifying the computation of relative changes of parameters to assess the effects of different exposure scenarios.

**Table 1:** Median and interquartile range (Q_1_–Q_3_) of pre-exposure PDA features for each exposure scenario.

	**FA**	**NaCl**	**HVO_**NOx**_**	**HVO_**NOx+PM**_**
T_1_ (ms)	151 (139 - 166)	154 (132 - 173)	155 (127 - 169)	146 (136 - 167)
T_12_ (ms)	147 (139 - 165)	145 (139 - 170)	148 (127 - 165)	141 (134 - 165)
T_13_ (ms)	300 (281 - 308)	299 (279 - 319)	298 (284 - 314)	293 (270 - 316)
T_14_ (ms)	432 (403 - 450)	433 (402 - 457)	426 (411 - 454)	426 (392 - 450)
T_15_ (ms)	538 (506 - 552)	535 (500 - 551)	531 (500 - 554)	528 (490 - 555)
W_1_ (ms)	178 (151 - 196)	173 (149 - 212)	178 (138 - 204)	168 (151 - 205)
A_12_ (%)	12.7 (9.8 - 18.3)	15.1 (10.8 - 21.7)	13.8 (9.5 - 23.0)	15.0 (10.8 - 21.8)
A_13_ (%)	16.6 (13.8 - 23.4)	23.2 (11.3 - 35.0)	16.1 (12.2 - 26.4)	18.8 (12.4 - 23.5)
A_14_ (%)	35.9 (29.9 - 46.1)	37.3 (26.6 - 51.9)	35.4 (26.1 - 42.9)	33.2 (29.1 - 42.3)
A_15_ (%)	50.5 (43.1 - 62.0)	51.2 (41.9 - 69.7)	52.1 (42.6 - 61.3)	50.8 (45.3 - 57.6)

**Figure 2:**
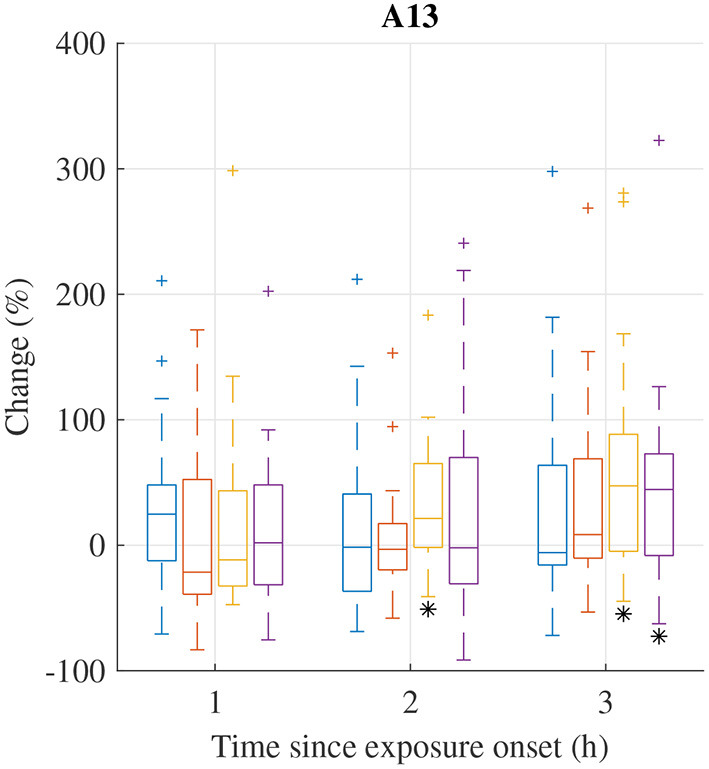
Median (central mark) and interquartile range (edges of box) of relative change $\Delta {\left(F_{i}\right)}^{t}$ of A_13_ during exposure scenario FA (blue), NaCl (red), HVO_NOx_(yellow), and HVO_NOx+PM_ (purple). Whiskers extend to 1.5 times the interquartile range and values outside this interval are marked by “+.” $\Delta {\left(F_{i}\right)}^{t}$ significantly different from zero according to the Wilcoxon signed rank test (p <0.05) are marked by (*).

The distribution of relative changes $\Delta {\left(F_{i}\right)}^{t}$ in A_13_ during each exposure scenario are displayed in Figure 2. A Wilcoxon signed rank test indicates that the increase in response to exposure scenario HVO_NOx_ at 2 and 3 h and exposure scenario HVO_NOx+PM_ at 3 h are significant (*p* < 0.05). However, individual differences are large.

Relative changes $\Delta {\left(F_{i}\right)}^{t}$ in PDA features from pre-exposure to 3 h after exposure onset are summarized in Table 2. In addition to the increases in A_13_, significant increases in A_12_ were observed during exposures NaCl and HVO_NOx_. The median of $\Delta {\left(F_{i}\right)}^{t}$ for A_13_ was considerably larger in exposure HVO_NOx+PM_ and HVO_NOx_ compared to FA, and the median of $\Delta {\left(F_{i}\right)}^{t}$ for A_12_ was considerably larger in exposure NaCl, HVO_NOx+PM_, and HVO_NOx_ compared to FA, however, the individual variation was large. The Friedman tests indicated that the differences between exposure types were not statistically significant.

**Table 2:** Median and interquartile range (Q_1_-Q_3_) of relative changes $\Delta {\left(F_{i}\right)}^{\text{3h}}$ in PDA features for each exposure scenario.

**Relative changes** $\Delta {\left(F_{i}\right)}^{\text{3h}}$ **(%)**								
$F_{i}$	**FA**		**NaCl**		**HVO**_**NOx**_		**HVO**_**NOx+PM**_	
T_1_	−5.2 (−7.7- 1.9)	n.s.	−2.4 (−10.2 - 6.5)	n.s.	−4.3 (−7.7 - 4.9)	n.s.	-2.9 (−11.3 - 8.6)	n.s.
T_12_	−2.8 (−9.3 - 2.1)	n.s.	-0.9 (-8.6 - 4.9)	n.s.	−5.1 (−10.7 - 2.7)	n.s.	−7.6 (−13.6 - 0.9)	n.s.
T_13_	−2.5 (−6.4 - 0.7)	n.s.	−1.5 (−9.1 - 2.7)	n.s.	−3.4 (−9.3 - 0.7)	n.s.	-3.9 (−10.1 - 2.9)	n.s.
T_14_	−1.6 (−5.9 - 1.0)	n.s.	−2.0 (−10.9 - 5.7)	n.s.	−4.1 (−9.6 - 2.6)	n.s.	−2.3 (−9.1 - 3.1)	n.s.
T_15_	−3.6 (−7.2 - 0.4)	n.s.	−3.2 (−8.6 - 4.1)	n.s.	−3.6 (−7.7 - 0.5)	n.s.	−2.5 (−9.1 - 1.6)	n.s.
W_1_	−2.1 (−10.2 - 6.3)	n.s.	-0.9 (−11.1 - 10.9)	n.s.	−2.3 (−9.7 - 9.1)	n.s.	-0.8 (−15.8 - 13.3)	n.s.
A_12_	11.9 (−21.2 - 63.6)	n.s.	22.5 (−10.2 - 108.4)	^*^	23.9 (-0.3 -82.1)	^*^	23.2 (−23.9 - 75.3)	n.s.
A_13_	-5.9 (−15.7 - 63.7)	n.s.	8.5 (−10.6 - 68.9)	n.s.	47.4 (-4.8 - 88.4)	^**^	44.4 (-8.2 - 72.8)	^*^
A_14_	−4.0 (−16.2 - 18.2)	n.s.	−3.2 (−6.9 - 55.7)	n.s	10.8 (−10.9 - 32.2)	n.s.	9.4 (-5.2 - 19.7)	n.s
A_15_	−4.8 (−11.7 - 8.2)	n.s.	−1.9 (−3.3 - 46.2)	n.s	8.7 (−11.8 - 19.4)	n.s.	6.6 (−5.4 - 16.2)	n.s

*$\Delta {\left(F_{i}\right)}^{\text{3h}}$ significantly different from zero according to the Wilcoxon signed rank test are indicated by*

* *p <0.05 or*

** *p <0.01, n.s. denotes not significant*.

### 4.3. Heart Rate Variability

The percentage of discarded RR intervals (mean±std) for each stage is 3.5 ± 6.6% (FA), 3.6 ± 6.7% (NaCl), 2.5 ± 3.4% 2.6 ± 4.8% (HVO_NOx_), and (HVO_NOx+PM_). These results suggest that few abnormal RR intervals are present in the ECG signal recordings in average. The number of analyzed RR intervals (mean±std) for each exposure type is 281 ± 52 (FA), 285 ± 47 (NaCl), 284 ± 42 (HVO_NOx_), and 282 ± 47 (HVO_NOx+PM_). Pre-exposure HRV statistics were not significantly different between exposure sessions, but a large range of responses was observed for every subject and feature individually (Table 3).

**Table 3:** Median and interquartile range (Q_1_-Q_3_) of pre-exposure HRV features for each exposure scenario.

	**FA**	**NaCl**	**HVO_**NOx**_**	**HVO_**NOx+PM**_**
NN (s)	1.044 (0.926 - 1.111)	0.996 (0.915 - 1.144)	1.035 (0.941 - 1.164)	1.017 (0.986 - 1.091)
SDNN (s)	0.058 (0.052 - 0.084)	0.064 (0.054 - 0.085)	0.062 (0.050 - 0.077)	0.061 (0.051 - 0.082)
SDSD (s)	0.050 (0.029 - 0.096)	0.042 (0.033 - 0.073)	0.055 (0.040 - 0.074)	0.055 (0.035 - 0.079)
rMSSD (s)	0.050 (0.029 - 0.096)	0.042 (0.033 - 0.073)	0.055 (0.040 - 0.074)	0.055 (0.036 - 0.079)
LF (s^2^)	0.001 (0.000 - 0.002)	0.001 (0.001 - 0.002)	0.001 (0.001 - 0.002)	0.001 (0.001 - 0.002)
HF (s^2^)	0.001 (0.001 - 0.003)	0.001 (0.000 - 0.003)	0.001 (0.001 - 0.002)	0.001 (0.001 - 0.002)
LF norm	0.543 (0.331 - 0.652)	0.538 (0.483 - 0.626)	0.534 (0.364 - 0.620)	0.547 (0.461 - 0.611)
HF norm	0.462 (0.354 - 0.670)	0.468 (0.385 - 0.524)	0.474 (0.387 - 0.648)	0.463 (0.395 - 0.543)
LF/HF	1.208 (0.493 - 1.846)	1.160 (0.921 - 1.624)	1.127 (0.562 - 1.606)	1.182 (0.855 - 1.549)

**Figure 3:**
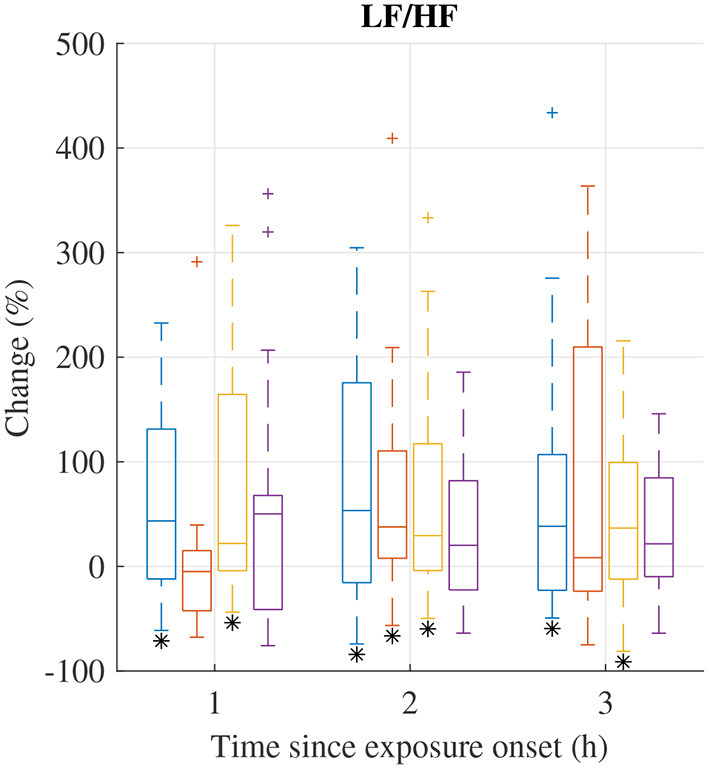
Median (central mark) and interquartile range (edges of box) of change $\Delta {\left(F_{I}\right)}^{t}$ of LF/HF during exposure scenario FA (blue), NaCl (red), HVO_NOx_(yellow), and HVO_NOx+PM_ (purple). Whiskers extend to 1.5 times the interquartile range and values outside this interval are marked by “+.” $\Delta {\left(F_{i}\right)}^{t}$ significantly different from zero according to the Wilcoxon signed rank test (p <0.05) are marked by (*).

The distribution of relative changes $\Delta {\left(F_{i}\right)}^{t}$ in LF/HF during each exposure scenario are displayed in Figure 3; increases were observed in several exposure sessions. A Wilcoxon signed rank test indicates that the increases during exposure scenario FA at 1h and 3 h, scenario NaCl at 2 h, and scenario HVO_NOx_ at 1, 2, and 3 h are significant (*p* < 0.05). However, individual differences are large.

Relative changes of HRV features from pre-exposure to 3 h after exposure onset are summarized in Table 4. I addition to the observed changes in LF/HF, heart rate was found to increase as indicated by the decrease in NN; the decrease in NN was significant for HVO_NOx_ but not for the other exposure scenarios. Further, LF was found to increase significantly during exposure scenario NaCl. The Friedman test show that no exposure type induce changes in HRV characteristics that were significantly different to the changes induced in the control session FA.

**Table 4:** Median and interquartile range (Q_1_–Q_3_) of relative changes $\Delta {\left(F_{i}\right)}^{\text{3h}}$ in HRV features for each exposure scenario.

**Relative changes** $\Delta {\left(F_{i}\right)}^{\text{3h}}$ **(%)**								
$F_{i}$	**FA**		**NaCl**		**HVO**_**NOx**_		**HVO**_**NOx+PM**_	
NN	−4.1 (−6.1 - 4.8)	n.s.	−4.3 (−15.8 - 6.2)	n.s.	-5.8 (−7.0 - −2.7)	^*^	−4.0 (−8.1 - 0.3)	n.s.
SDNN	−5.4 (−15.9 - 29.0)	n.s.	−6.8 (−21.4 - 10.7)	n.s.	−12.0 (−18.2 - 21.9)	n.s.	0.4 (−21.1 - 27.6)	n.s.
SDSD	−10.0 (−26.6 - 21.3)	n.s.	-14.5 (−26.5 - 14.1)	n.s.	−19.6 (−28.0 - −3.8)	n.s.	−4.9 (−20.3 - 21.0)	n.s.
rMSSD	−10.0 (-26.6 - 21.3)	n.s.	−14.4 (−26.5 - 14.1)	n.s.	-19.6 (−27.9 - −3.8)	n.s.	−4.9 (-20.3 - 21.1)	n.s.
LF	13.7 (−19.8 - 105.9)	n.s.	61.3 (−39.6 - 116.5)	⋆	−8.2 (−50.5 - 28.1)	n.s.	31.0 (−30.1 - 138.0)	n.s.
HF	1.8 (−40.2 - 43.0)	n.s.	-11.9 (−37.2- 31.1)	n.s.	-25.4 (−60.1 - 10.3)	n.s.	15.6 (-34.4 - 61.7)	n.s.
LF norm	10.6 (-11.0 - 53.6)	n.s	0.4 (−11.5 - 39.1)	n.s.	12.7 (−5.5 - 43.9)	n.s	10.7 (−5.3 - 42.2)	n.s.
HF norm	−16.4 (−29.7 - 14.1)	n.s.	−4.1 (−41.1 - 12.6)	n.s.	-15.5 (−30.4 - 8.6)	n.s.	−6.9 (−23.4 - 5.4)	n.s.
LF/HF	38.4 (−22.9 - 106.9)	⋆	8.4 (−23.7 - 209.8)	n.s.	36.7 (-12.2 - 99.4)	⋆	21.6 (−9.8 - 84.7)	n.s.

*$\Delta {\left(F_{i}\right)}^{\text{3h}}$ significantly different from zero according to the Wilcoxon signed rank test are indicated by*

* p <0.05 or **p <0.01, n.s. denotes not significant.

### 4.4. Pulse Transit Time

The percentage of discarded PTT intervals (mean±std) for each stage is 4.2 ± 9.5% (FA), 4.8 ± 12.0% (NaCl), 6.6 ± 16.5% (HVO_NOx_), and 2.6 ± 5.9% (HVO_NOx+PM_). The results remain heterogeneous, with several recordings concerned by a percentage of discarded PTT intervals superior to 15% (2 for FA, 4 for NaCl, 4 for HVO_NOx_, and 1 for HVO_NOx+PM_). However, the number of analyzed PTT intervals in each 5-min recording (mean±std) 284 ± 54 (FA), 287 ± 55 (NaCl), 275 ± 60 (HVO_NOx_), and 288 ± 46 (HVO_NOx+PM_) is considered sufficient for analysis.

Pre-exposure PTT features were not significantly different between exposure sessions, but a large range of responses was observed for every subject and feature individually (Table 5).

**Table 5:** Median and interquartile range (Q_1_-Q_3_) of pre-exposure PTT features for each exposure scenario.

	**FA**	**NaCl**	**HVO_**NOx**_**	**HVO_**NOx+PM**_**
PTT (s)	0.164 (0.156 - 0.175)	0.162 (0.157 - 0.173)	0.165 (0.156 - 0.169)	0.159 (0.144 - 0.167)
SDPTT (s)	0.006 (0.005 - 0.007)	0.006 (0.005 - 0.006)	0.006 (0.005 - 0.007)	0.005 (0.004 - 0.006)
SDSD^PTT^ (s)	0.006 (0.005 - 0.008)	0.006 (0.005 - 0.008)	0.006 (0.005 - 0.007)	0.005 (0.004 - 0.007)
rMSSD^PTT^ (s)	0.006 (0.005 - 0.008)	0.006 (0.005 - 0.008)	0.006 (0.005 - 0.007)	0.005 (0.004 - 0.007)

**Figure 4:**
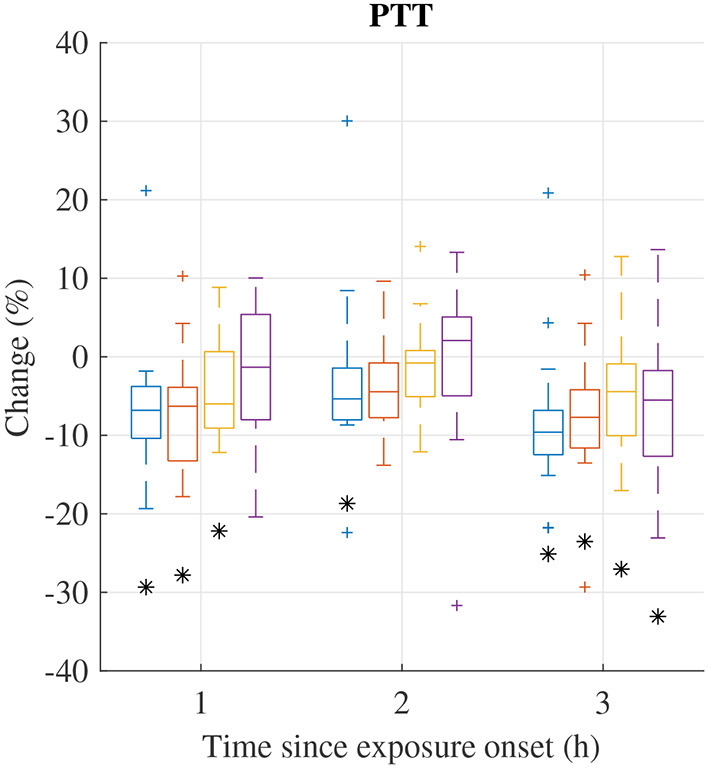
Median (central mark) and interquartile range (edges of box) of change $\Delta {\left(F_{i}\right)}^{t}$ of mean PTT during exposure scenario FA (blue), NaCl (red), HVO_NOx_(yellow), and HVO_NOx+PM_ (purple). Whiskers extend to 1.5 times the interquartile range and values outside this interval are marked by “+.” $\Delta {\left(F_{i}\right)}^{t}$ significantly different from zero according to the Wilcoxon signed rank test (p <0.05) are marked by (*).

The distribution of relative changes $\Delta {\left(F_{i}\right)}^{t}$ in PTT during each exposure scenario are displayed in Figure 4. Significant decreases were observed during all exposure sessions. However, individual differences were large.

Relative changes $\Delta {\left(F_{i}\right)}^{t}$ of PTT features from pre-exposure to 3 h after exposure onset are summarized in Table 6. In addition to the decrease in PTT, a significant decreases were observed for rMSSD^PTT^ and SDSD^PTT^ during exposures FA and HVO_NOx_. The Friedman test show that no exposure type induces changes in PTT characteristics that was significantly different to the changes induced in the control session FA.

**Table 6:** Median and interquartile range (Q_1_–Q_3_) of relative changes $\Delta {\left(F_{i}\right)}^{\text{3h}}$ in PTT features for each exposure scenario.

**Relative changes** $\Delta {\left(F\right)}^{\text{3h}}$ **(%)**								
F	**FA**		**NaCl**		**HVO**_**NOx**_		**HVO**_**NOx+PM**_	
PTT	−9.6 (−12.5 - −6.8)	^**^	−7.7 (−11.6 - −4.2)	^**^	−4.4 (−10.1 - −0.9)	^**^	−5.5 (−12.7 - −1.8)	⋆
SDPTT	−13.5 (−27.6 - −6.8)	n.s	−0.1 (−30.4 - 35.8)	n.s.	−17.4 (−24.5 - 15.0)	n.s.	−1.8 (−24.0 - 20.6)	n.s.
rMSSD^PTT^	−20.2 (−34.1 - 2.3)	^*^	−14.1 (−31.2 - 25.5)	n.s.	−25.0 (−27.9 - −8.9)	^*^	−11.2 (−24.6 - 15.2)	n.s.
SDSD^PTT^	−20.2 (−34.1 - 2.3)	^*^	−14.1 (−31.3 - 25.5)	n.s.	−25.0 (−27.9 - −8.9)	^*^	−11.2 (-24.6 - 15.2)	n.s.

*$\Delta {\left(F_{i}\right)}^{\text{3h}}$ significantly different from zero according to the Wilcoxon signed rank test are indicated by*

* *p <0.05) or*

** *p <0.01, n.s. denotes not significant*.

## 5. Discussion

The present results show that PPG pulse characteristics, PTT and HRV change during the chamber exposure sessions. However, the individual variation is large and differences between exposure sessions were not statistically significant.

The methodology applied to analyze changes in PPG pulse characteristics in the present study has previously been used to assess differences in autonomic reactivity to mental stress in patients with mental depressive disorder and healthy controls (Kontaxis et al., 2021); the methodology was first proposed for robust extraction of PPG pulse features in the context of surrogate baroreflex sensitivity measurements (Lázaro et al., 2019). Each PPG pulse is decomposed into a main wave and several reflected waves; the waves are assumed to be symmetrical but no specific shape is assumed. Several other methods have been proposed for PPG pulse decomposition based on fitting of Gaussian functions (Rubins, 2008; Liu et al., 2013; Wang et al., 2013; Couceiro et al., 2015), Rayleigh functions (Goswami et al., 2010), and Log-Normal functions (Huotari et al., 2011). In contrast to Lázaro et al. (2019) where the waves are fitted sequentially, these methods are based on joint fitting a sum of two (Goswami et al., 2010) to five waves (Huotari et al., 2011; Wang et al., 2013; Couceiro et al., 2015). Whereas (Kontaxis et al., 2021) decomposed the PPG pulses into three waves, five waves were used in the present study. We obtained equally robust estimates of the amplitude and timing for the 4th and 5th decomposed waves as for the 2nd and 3rd wave (results not shown).

Pulse decomposition analysis is based on the concept of arterial pressure pulses originating from the left ventricle being reflected in the arterial tree. The first decomposed wave resulting from PDA corresponds to the primary pressure pulse and the following decomposed waves results from these reflections. Baruch (2019) argues that two distinct central pressure pulse reflection sites contributes most; one in the region of the renal arteries and one beyond the bifurcation of the iliac arteries. Reflections from these sites are assumed to correspond to the second and third decomposed wave, respectively, and the fourth and fifth decomposed waves are assumed to be caused by re-reflections between these sites. Stiffer arteries are assumed to increase the propagation of the arterial pulse and enhance the speed and magnitude of the reflected waves.

The PPG pulse feature A_13_ quantifies relative amplitude loss in the second reflected wave. The increase in A_13_ during exposure HVO_NOx_ and HVO_NOx+PM_ may reflect changes in vascular tone induced by the exposure to HVO exhaust, since similar increases in A_13_ were not observed during the control session FA. The increases in LF/HF indicating increased sympathetic tone observed during the control scenario FA suggest that the experiment induces stress; such increases are also observed during the other exposure scenarios. Other possible reasons for the observed changes in LF/HF is that the time of the day changes during the experiment; circadian variation in short-term HRV indexes obtained from healthy subjects have previously been reported (Bilan et al., 2005). The decrease in PTT observed during the control scenario FA suggest that changes in vascular tone are induced during the experiment; similar decreases in PTT are observed during exposure scenarios NaCl, HVO_NOx_, and HVO_NOx+PM_. The decrease in PTT most likely reflect increases in PWV caused by increased sympathetic tone; such increases have previously been shown in response sympathoexcitatory maneuvers such as cold pressure test, lower body negative pressure, and orthostasis (Nardone et al., 2020).

It should be noted that whereas the decrease in PTT is associated to increased arterial stiffness, the increase in A_13_ quantifying a decreased magnitude of the reflected wave is rather assumed to be associated to the opposite. However, vascular smooth muscle tone can also can influence wave reflection characteristics independent of artery stiffness. Kelly et al. (2001) showed that systemic administration of the arterial vasodilator nitroglycerin reduced the augmentation index quantifying the magnitude of the reflected wave, but had little effect on the pulse wave velocity. Since the increase in A_13_ was observed both during exposure HVO_NOx+PM_ and HVO_NOx_, it may be associated to inhalation of nitric oxides rather than particulate matter. NO is known as a vasodilator (Levine et al., 2012), and inhalation of NO has been proposed as a selective pulmonary vasodilator (Ichinose et al., 2004). The levels of inhaled NO during the HVO exposures in the present study are close to therapeutic levels of inhaled NO (5–80 ppm) (Yu et al., 2019). On the other hand, long-term exposure to NO_2_ is linked to increased arterial stiffness; (Lenters et al., 2010) reported increases in augmentation index and pulse wave velocity associated to increases in NO_2_ study involving 729 young individuals. However, no such association was found in subsequent studies on short-term NO_2_ exposure (Mehta et al., 2014; Wu et al., 2016).

Transient increases in arterial stiffness have previously been reported in response to exposure to diluted diesel exhaust (Lundbäck et al., 2009) as well as diluted wood smoke (Unosson et al., 2013). It should be noted that the PM concentrations in those studies were larger, ≈350 and ≈314μ*gm*^−3^ of PM_2.5_, respectively. Further, in contrast to the present study, where the participants were at rest throughout the exposure session, the participants in those studies were exercising moderately and thereby possibly increasing the exposure. The present study was conducted on a study population of healthy young and middle-aged adults. Our results are consistent with the results presented in Mills et al. (2011a), where brief exposure to diluted diesel exhaust (≈300μ*gm*^−3^) did not alter HRV in healthy volunteers. A study population consisting of older and overweight adults may have given a different result; (Hemmingsen et al., 2015) found that HRV significantly decreased during 5 h exposure to real-life levels of particles from urban streets in a study population of overweight, middle-aged and elderly adults.

### 5.1. Limitations

The main limitation of the present study is the small study population. Considering that the individual variation of changes in PPG pulse characteristics, HRV and PTT in response to the exposures is very large, a larger study population is desired. It should be noted, however, that including a large number of participants in a chamber exposure study is associated with major difficulties. Each volunteer has to spend half a day at four different occasions for the experiments. Further, the exposure chamber can fit a maximum of four study participants at the time. Another limitation is the lack of comparison to reference clinical standard measurements of pulse wave velocity and pulse wave reflection. Previous studies on air pollution and arterial stiffness are based on planation tonometry, and it should be noted that the results of PPG based assessment of pulse wave velocity and pulse wave reflections presented in this study are not directly comparable to the results in the previous studies.

## 6. Conclusions

The aim of the present study was to characterize changes in PPG pulse morphology, HRV, and PTT in response to exposure to diluted HVO exhaust in a randomized double-blinded cross-over study. Significant increases in LF/HF and significant decreases in PTT were observed in all exposure scenarios. Further, the PDA feature A_13_, reflecting vascular compliance, increased significantly in the HVO_NOx_ and HVO_NOx+PM_ exposure sessions but not in the FA and NaCl sessions. The individual variation of the changes in PDA, HRV, or PTT features during the exposures were large, and no significant differences in between exposure sessions were found.

## Data Availability Statement

The raw data supporting the conclusions of this article will be made available by the authors, without undue reservation.

## Ethics Statement

The study was conducted in accordance with the Declaration of Helsinki. It was reviewed and approved by the Swedish Ethical Review Authority (registration no. 2019-03320). All participants gave their informed consent before the experiment.

## Author Contributions

LG, KD, AG, AW, and FS contributed to the study design and data acquisition. YM-D and FS performed the data analysis and drafted the manuscript. MA contributed to the clinical interpretation of the results. AG and AW conceived the original idea for the exposure project and acquired funding. AW coordinated the exposure project. All authors contributed to the final version of the manuscript.

## Funding

This work was supported by the Swedish Research Council for Sustainable Development (grant FORMAS2016-00824), the Swedish Research Council (grant VR2019-04272), and the Crafoord Foundation (grant 20200605).

## Conflict of Interest

The authors declare that the research was conducted in the absence of any commercial or financial relationships that could be construed as a potential conflict of interest.

## Publisher's Note

All claims expressed in this article are solely those of the authors and do not necessarily represent those of their affiliated organizations, or those of the publisher, the editors and the reviewers. Any product that may be evaluated in this article, or claim that may be made by its manufacturer, is not guaranteed or endorsed by the publisher.
